# Symmetry Breaking in the Lowest-Lying Excited-State of CCl_4_: Valence Shell Spectroscopy in the 5.0–10.8 eV Photon Energy Range

**DOI:** 10.3390/molecules29235619

**Published:** 2024-11-27

**Authors:** Luiz V. S. Dalagnol, Sarvesh Kumar, Alessandra S. Barbosa, Umma S. Akther, Nykola C. Jones, Søren V. Hoffmann, Márcio H. F. Bettega, Paulo Limão-Vieira

**Affiliations:** 1 Departamento de Física, Universidade Federal do Paraná, Caixa Postal 19044, Curitiba 81531-980, Paraná, Brazil; lvsd15@fisica.ufpr.br (L.V.S.D.); alessandra@fisica.ufpr.br (A.S.B.); 2 Atomic and Molecular Collisions Laboratory, CEFITEC-Centre of Physics and Technological Research, Department of Physics, NOVA School of Science and Technology, Universidade NOVA de Lisboa, 2829-516 Caparica, Portugal; s.kumar@campus.fct.unl.pt (S.K.); u.akther@campus.fct.unl.pt (U.S.A.); 3 Lawrence Berkeley National Laboratory, Chemical Sciences Division, One Cyclotron Road, Berkeley, CA 94720, USA; 4 ISA, Department of Physics and Astronomy, Aarhus University, Ny Munkegade 120, DK-8000 Aarhus, Denmark; nykj@phys.au.dk (N.C.J.); vronning@phys.au.dk (S.V.H.)

**Keywords:** carbon tetrachloride, cross-sections, theoretical calculations, spectroscopy

## Abstract

We report absolute high-resolution vacuum ultraviolet (VUV) photoabsorption cross-sections of carbon tetrachloride (CCl_4_) in the photon energy range 5.0–10.8 eV (248–115 nm). The molecular spectrum and electronic structure have been comprehensively investigated together with quantum chemical calculations, providing geometries, bond lengths, vertical excitation energies and oscillator strengths. The major electronic excitations have been assigned to valence and Rydberg transitions which are also accompanied by vibrational excitation assigned to degenerate stretching, $v_{3}^{\prime }\left(t_{2}\right)$ and degenerate deformation $v_{4}^{\prime }\left(t_{2}\right)$ modes. The rather complex nuclear dynamics along the degenerate deformation mode, $v_{4}^{\prime }\left(t_{2}\right)$, have been thoroughly investigated by Time-Dependent Density Functional Theory (TD-DFT) method. The relevant Jahn–Teller distortion operative within the lowest-lying electronic excited-state is shown here for the first time in order to yield a weak absorption feature at 6.156 eV. Further calculations on the potential energy curves for the singlet excited-states along the C–Cl stretching coordinate show the relevance of efficient C–Cl bond excision.

## 1. Introduction

Carbon tetrachloride, also known as tetrachloromethane (CCl_4_), is a chemical solvent which has been widely used as a dry cleaning solvent in industrial applications and also in reactive ion etching [1,2,3]. It is relevant as an anthropogenic atmospheric chemical compound and is recognised as a greenhouse gas [4,5,6]; it can also undergo photolysis with chlorine atoms diffusing to higher altitudes and therefore posing a threat to the ozone layer [3,7]. Although it has been phased out under the Montreal Protocol, it is still used as a contained feedstock for hydrofluorocarbon production [4]. In addition, for research applications, CCl_4_ has been used in low-energy electron-induced processes, viz. dissociative electron attachment (DEA), to set the energy scale and resolution in these experiments. This is achievable from the relevant Cl^−^ yield at virtually no electron energy, i.e., ~0 eV [8,9] (and references therein). Therefore, the main motivation for photon absorption cross-section data for CCl_4_ is the current need to provide an updated and reliable wealth of information for databases that can be used to model such interactions and applications of this molecule mainly in atmospheric modelling, while providing a detailed characterisation of its electronic state spectroscopy.

The CCl_4_ molecule has been investigated by gas-phase vacuum ultraviolet (VUV) absorption in energy ranges from 5.0 to 10.8 eV (115 to 250 nm) [10,11,12,13] and from 6 to 250 eV [14], using fluorescence spectroscopy [12,15], infrared and Raman spectroscopy [16,17,18] and photodissociation with quantum yields [12,19,20,21,22]. The experimental lowest-lying ionisation energies have been reported by He(I) and He(II) photoelectron spectroscopies [23,24,25,26,27] and Multi-Reference Configuration Interaction (MRCI) studies [28]. Also relevant are the scattering dynamics for electron collisions from CCl_4_ [29,30,31,32,33,34,35,36,37], theoretical studies on vibrational spectroscopy [38], valence shell excitation [39] and the stability of CCl_4_ ions [3].

The present work deals with a complete description of the electronic state spectroscopy of CCl_4_ in the energy range from 5.0 to 10.8 eV by combining high-resolution VUV photoabsorption experiments from a synchrotron light source together with Time-Dependent Density Functional Theory (TD-DFT/PBE0/aug-cc-pVDZ) calculations on the electronic excitation energies and oscillator strengths (in the length gauge) for the lowest-lying neutral states. Within the complex nuclear dynamics of the lowest-lying excited-state, the relevant Jahn–Teller distortion in the instability of CCl_4_ has been investigated by providing potential energy curves on the degenerate deformation mode, $v_{4}^{\prime }\left(t_{2}\right)$, while allowing all the atoms to relax following this mode.

## 2. Structure and Properties of Carbon Tetrachloride

**Figure 1:**
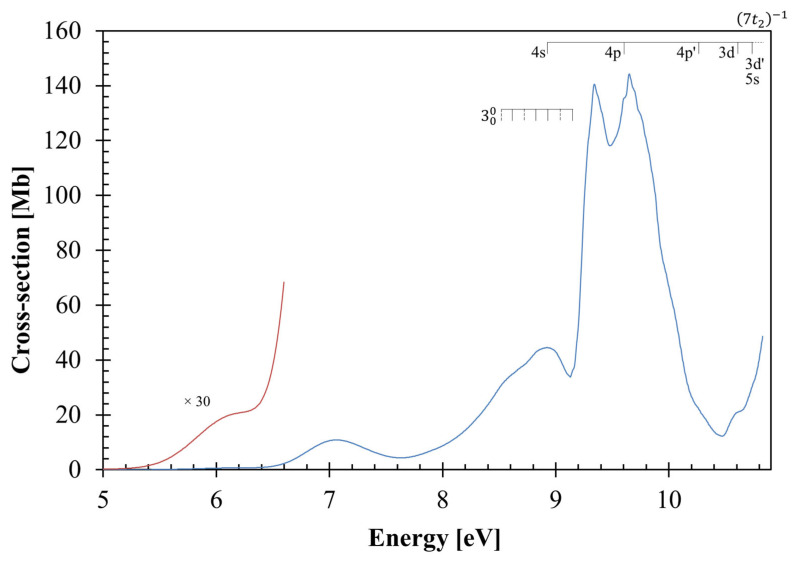
The high-resolution photoabsorption spectrum of carbon tetrachloride in the 5.0–10.9 eV photon energy range. Dashed lines are tentative assignments. See the text for details.

**Figure 2:**
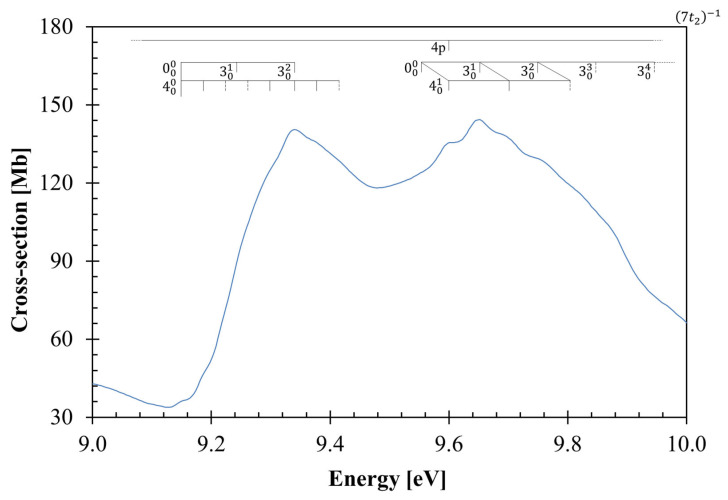
Detail of the photoabsorption spectrum of carbon tetrachloride in the 9.0–10.0 eV photon energy range. Dashed lines are tentative assignments. See the text for details.

The neutral ground-state (*T*_d_) and the cationic ground-state (*C*_1_) geometries of CCl_4_ obtained at the DFT/PBE0/aug-cc-pVDZ level, together with their bond lengths (Å) and bond angles (°), are listed in the Supporting Material in Figures S1 and S2. The calculated outermost electronic configuration of the X~A11 ground-state (Figure S1) is as follows: …(14a_1_)^2^ (3a_2_)^2^ (15a_1_)^2^ (8b_1_)^2^ (8b_2_)^2^ (9b_2_)^2^ (4a_2_)^2^ (9b_1_)^2^. In Figure S3, we show a representation of molecular orbitals with the highest occupied molecular orbital (HOMO), 9b_1_ and the Cl 3p lone pair orbital $\left(n_{Cl}\right)$. The (HOMO-1), 4a_2_, the (HOMO-2), 9b_2_, the (HOMO-6), 3a_2_, and the (HOMO-7), 14a_1_, are also shown in Figure S3 and have $\left(n_{Cl}\right)$ characters. The other MOs from which electrons can be excited, namely (HOMO-3), 8b_2_, (HOMO-4), 8b_1_, and (HOMO-5), 15a_1_, are $n_{Cl}/\sigma_{CCl}$ in character. A representation of a selection of carbon tetrachloride molecular orbitals at the DFT/PBE0/aug-cc-pVTZ level according to the *C*_2v_ point group is also shown in Figure S4. The main absorption features in Figure 1 and Figure 2 are due to electronic excitations from such MOs to valence and Rydberg orbitals, with the calculated dominant excitation energies and oscillator strengths listed in Table 1, while the complete calculated electronic transitions are in Table S1.

The photoabsorption spectrum in Figure 1, together with an expanded view of the measured cross-sections between 9 and 10 eV in Figure 2, show some fine structures which have been assigned to vibronic transitions, with the two main vibrational modes assigned according to the experimental infrared spectroscopy of Wallington et al. [18]. Additional information has been obtained from the calculation of the harmonic frequencies and assignment in Tables S3 and S4. The main modes have been assigned based on the energies (and wavenumbers) in the neutral electronic ground-state to 0.096 eV (776 cm^−1^) for the degenerate stretching $v_{3}^{\prime \prime }\left(t_{2}\right)$ and 0.039 eV (314 cm^−1^) for the degenerate deformation $v_{4}^{\prime \prime }\left(t_{2}\right)$ modes (see Table S3). The harmonic frequencies of the cationic electronic ground-state have also been considered in the assignments of the Rydberg character of the electronic transitions with the major contributions of the C–Cl stretching/deformation, $v_{6}^{\prime }\left(a\right)$ (0.059 eV, 476.2 cm^−1^), and the asymmetric stretching, $v_{9}^{\prime }\left(a\right)$ (0.115 eV, 929.6 cm^−1^), modes (see Table S4 and Section 3.4). It is important to note that neutral electronic first excited-state harmonic frequencies were not obtained due to prompt C–Cl bond breaking upon geometry optimisation.

The structures assigned throughout the photoabsorption spectrum appear in the notation format $X_{m}^{n}$, with *m* and *n* indicating the initial and final vibrational states for the vibronic structure (*X*). The tentative assignment of the Rydberg orbitals has been performed based on the quantum defects and the adiabatic ionisation energy (IE)_ad_ from the photoelectron data of Bassett and Lloyd [26] to be IE_2_ = 12.27 eV (7t_2_)^−1^. No attempt was made to assign Rydberg series converging to (2t_1_)^−1^ (IE_1_=11.47 eV) because such an electronic transition is dipole forbidden within the molecule’s *T*_d_ group symmetry, while the Rydberg series converging to higher ionisation energies lie outside the energy range of the present photoabsorption spectrum.

## 3. Discussion

Figure 1 shows the photoabsorption spectrum of carbon tetrachloride in the energy range 5.0 to 10.8 eV (in the energy range from 4 to 5 eV there is no absorption), while Figure 2 shows an enlarged section from 9 to 10 eV. The most representative calculated vertical excitation energies and oscillator strengths are listed in Table 1 together with the assignment of the different absorption features related to the valence and Rydberg transitions. The comparison with the experimental data shows a good level of agreement within ±7%. The CCl_4_ molecular orbitals participating in the dominant electronic excitations are depicted in Figure S3, with electrons being promoted from the $n_{Cl}/\sigma_{CCl}\;$ to the $\sigma_{CCl}^{*}$ antibonding or to the Rydberg MO orbitals (Table 1). The vertical excitation energy of the lowest absorption band at 6.156 eV (Figure 1) is not reproduced by the calculations in the Born–Oppenheimer approximation. However, we have comprehensively investigated the nuclear dynamics along the degenerate deformation mode, $v_{4}^{\prime }\left(t_{2}\right)$, to obtain its vertical value (see Section 3.1).

Table 2 and Table 3 contain the proposed vibrational assignments in the photon energy range 8.5−10.0 eV and the different Rydberg series, converging to a (7t_2_)^−1^ A~T22 ionic electronic first excited-state (Section 3.4). The absorption bands above 8 eV show a fine structure assigned to the degenerate stretching, $v_{3}^{\prime }\left(t_{2}\right)$, and the degenerate deformation $v_{4}^{\prime }\left(t_{2}\right)$ modes (Figure 1 and Figure 2), together with combinations of these. Note that $v_{4}^{\prime }\left(t_{2}\right)$ is also known to be a Fermi resonance of the symmetric C–Cl stretching $v_{1}^{\prime }\left(a_{1}\right)$ and the degenerate deformation $v_{2}^{\prime }\left(e\right)$ modes; these modes are dipole forbidden within the molecule’s *T*_d_ group symmetry.

### 3.1. The 5.0–6.5 eV Photon Energy Range

**Figure 3:**
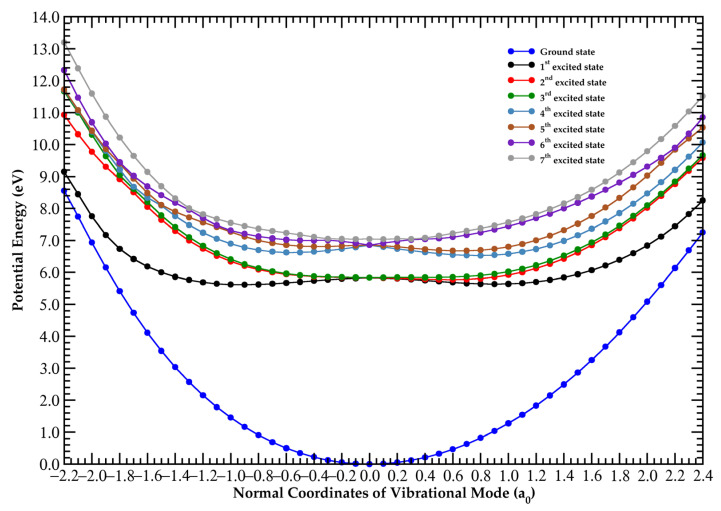
PECs for the seven lowest-lying excited singlet states of carbon tetrachloride plotted following the degenerate deformation mode $v_{4}^{\prime }\left(t_{2}\right)$ (in a_0_ units). The calculations were performed at the TD-DFT/PBE0/aug-cc-pVDZ level of theory in the *C*_1_ symmetry group. See the text for details.

Upon electronic excitation from the degenerate *T*_d_ symmetry ground-state, the CCl_4_ molecule distorts, removing the degeneracy and forming a lower symmetry system. Therefore, the lowest-lying valence transition at 6.156 eV, with a local cross-section of 0.68 Mb, results from a symmetry breaking related to the Jahn–Teller distortion of the carbon tetrachloride first excited-state. This is not obtained from the present calculations where the molecular frame is frozen during electronic excitation. To further our knowledge on the underlying nuclear dynamics governing such an electronic transition, we have comprehensively investigated the potential energy curves (PECs) for the seven lowest-lying excited singlet states plotted along the degenerate deformation mode, $v_{4}^{\prime }\left(t_{2}\right)$ (in a_0_ units) (see Figure 3). The calculations were performed at the TD-DFT/PBE0/aug-cc-pVDZ level of theory in the *C*_1_ symmetry group. An inspection of Figure 3 shows that all the excited-states are degenerate at the equilibrium normal coordinate and are split as one moves away from the reference position, i.e., 0 a_0_. The triply degenerate lowest-lying excited-states give rise to a rather shallow PEC (black dots in Figure 3) at ~−0.9 a_0_ (and even at around 1.0 a_0_), which corresponds to the first excited singlet–singlet electronic transition with a vertical excitation energy of 5.833 eV in reasonable agreement with the 6.156 eV experimental value (Figure 1). It is assigned to the promotion of an electron from the lone pair orbital $n_{Cl}$ to a $\sigma_{CCl}^{*}$ antibonding molecular orbital.

We have obtained the PECs along the C1–Cl_3_ and Cl_2_–C1–Cl_3_ stretching coordinates, with the results shown in Figures S5 and S6. An abstraction of either a single or two chlorine atoms, already at energies ≳ 6 eV, can occur via access to the lowest-lying excited electronic states within the Franck–Condon region. In Figure S5, we show that upon electronic excitation from the ground-state, the triply degenerate excited-states at the equilibrium geometry of the neutral molecule are accessed. As the nuclear wave packet evolves, a single Cl atom can be formed either above the asymptotic limit with an excess of kinetic energy (the double degenerate first and second excited-states, black and red dots, respectively) or can just simply tunnel through the barrier as long as the wave function amplitude is non-zero (third excited-state, green dots). However, when two C–Cl bonds are stretched, Cl + Cl formation may be not operative across the energy range from the ground-state up to 8 eV (Figure S6). This is due to the potential energy curves’ deepness that is accessible within the Franck–Condon region, which can hardly lead to the asymptotic limits. The geometries of CCl_4_ in the ground- and first excited-state together with their bond lengths in Å and bond angles in (°) are shown in Figures S1 and S7. For the first electronic excited-state, geometry optimisation leads to prompt C1–Cl_2_ and C1–Cl_4_ bond excisions with a Cl_5_–C1–Cl_3_ angle of ~66° (Figure S7), while in the neutral ground-state the angle is ~110° (Figure S1). This shows the relevant C–Cl bending mechanism that may be operative within the dichloromethylene radical, CCl_2_^•^, which is in assertion with fluorescence spectroscopy [21,22] and the mass spectrometric formation of a stable but weak signal of CCl_2_^−^ in electron transfer experiments [40].

### 3.2. The 6.5–9.2 eV Photon Energy Range

The second absorption band centred at 7.06(1) eV with a maximum cross-section of 10.87 Mb in Figure 1 is assigned to the valence excitation of the chlorine lone pair with σ MO $n_{Cl}/\sigma \left({15a}_{1},{8b}_{1},{8b}_{2}\right)$ to the σ*(16a_1_) antibonding orbital, σ*16a1←nCl/σ, 1T21←X~A11, with an oscillator strength *f*_L_ = 0.0202 (Table 1). The energy position of the band has also been reported in the vacuum ultraviolet absorption spectra of Causley and Russell [11] at 7.041 eV, Russell et al. [13] at 7.042 eV and Ho [14] at 7.08 eV, while Watanabe and Takahashi’s [39] theoretical study of valence shell excitation by high-energy electron impact reported a vertical value at 7.09 eV.

The next vertical electronic transition peaking at 8.92(9) eV, and a local cross-section of 44.48 Mb, is also valence in character. It is assigned to the promotion of the chlorine lone pair $n_{Cl}\left({{14a}_{1},3a}_{2}\right)$ and mixed MOs $n_{Cl}/\sigma \left({15a}_{1},{8b}_{2},{8b}_{1}\right)$ to the $\sigma^{*}\left({17a}_{1},{10b}_{2},{10b}_{1}\right)$ antibonding orbitals, σ*←σ+σ*←nCl/σ, 3T21←X~A11, with *f*_L_ = 0.1254 (Table 1). Causley and Russell [11] report the absorption feature at 8.859 eV, Russell et al. [13] at 8.852 eV and Watanabe and Takahashi [39] at 8.87 eV. The $0_{0}^{0}$ origin assigned at 8.52(1) eV (Table 2 and Figure 1) shows broad and weak features which are reminiscent of the pre-dissociative character of the absorption band. The vibrational assignment is due to the degenerate stretching $v_{3}^{\prime }\left(t_{2}\right)$ mode, with an average value of 0.104 eV (Table 2). Note in Figure 1 and Table 2 that some of the weak vibrational features’ tentative positions are marked as dashed lines.

### 3.3. The 9.2–10.8 eV Photon Energy Range

The absorption band with a vertical value of 9.343 eV and a magnitude of 140.41 Mb is due to a mixed valence and Rydberg character transition, 4s←nCl/σ+σ*←nCl+4T21←X~A11, with *f*_L_ = 0.0358 (Table 1). Although the calculated oscillator strength is one order of magnitude lower than the value for the next transition (Table 1), the absorption band appears rather intense since it may borrow a relevant magnitude from the proximity to the most intense electronic excitation. Another relevant aspect from the calculation pertains to the 4s Rydberg contribution just being a mere 4%, yet the experimental value is suggested at 8.92(9) eV from the quantum defect estimation (see below). The $0_{0}^{0}$ origin of the band is at 9.14(7) eV and is mainly accompanied by the excitation of the degenerate deformation mode, $v_{4}^{\prime }\left(t_{2}\right)$, and the degenerate stretching, $v_{3}^{\prime }\left(t_{2}\right)$, mode, with mean energy values of 0.038 and 0.098 eV (Table 2). Some of the proposed $v_{4}^{\prime }\left(t_{2}\right)$ vibrational assignments in Figure 2 appear as dashed lines due to their weak intensity nature. Moreover, the lowest-lying *n* = 4 member of the *ns* Rydberg series converging to (7t_2_)^−1^ A~T22 appears at 8.92(9) eV and may also contain contributions of two quanta from the degenerate stretching $v_{3}^{\prime }\left(t_{2}\right)$ mode superimposed on the 3T21←X~A11 valence transition (see Section 3.4).

The absorption band with the highest cross-section of carbon tetrachloride peaks at 9.652 eV, with a magnitude of 144.31 Mb (Figure 1 and Figure 2), is assigned in Table 1 to a mixed valence and Rydberg character 4s←nCl/σ+4p/4p′←nCl+σ*←nCl, 5T21←X~A11 transition with the highest oscillator strength *f*_L_ = 0.2174. The quantum defect obtained for the feature in this absorption band suggests the presence of only an *n* = 4p member of the Rydberg series converging to the ionic electronic first excited-state, (7t_2_)^−1^ A~T22 (see Section 3.4). The $0_{0}^{0}$ origin band is at 9.55(2) eV and shows four quanta of the degenerate stretching $v_{3}^{\prime }\left(t_{2}\right)$ mode in combination with the degenerate deformation mode $v_{4}^{\prime }\left(t_{2}\right)$ (Figure 2 and Table 2). In Figure 2, the high-energy side of the absorption band exhibits tentative assignments of weak features and is marked as dashed lines.

Above 10 eV, the photoabsorption energy range comprises two other electronic excitations (Figure 1) which are assigned to Rydberg transitions 4s←nCl/σ+4p/4p′←nCl, 6T21←X~A11 and 4p/4p′←nCl/σ+3d/3d′←nCl, 7T21←X~A11. The former band has a vertical excitation energy at 10.26(4) eV, and a cross-section value of 22.16 Mb, while for the latter we are not able to give its maximum value because it lies beyond the energy range of the present absorption spectrum (Table 1).

### 3.4. Rydberg Transitions

The photoabsorption energy values, quantum defects (*δ*) and assignments of the Rydberg series converging to (7t_2_)^−1^ A~T22 of carbon tetrachloride are listed in Table 3. The energy features’ positions have been tested from the Rydberg formula, $E_{n}=IE-R/{\left(n-\delta \right)}^{2}$, where *IE* is the ionisation energy of a given MO, *n* is the principal quantum number of the Rydberg orbital of energy *E*_n_, *R* is the Rydberg constant (13.61 eV) and *δ* is the quantum defect resulting from the penetration of the Rydberg orbital into the core. The Rydberg character of the absorption features is only noted above 8.5 eV, because the electronic excitation from CCl_4_ neutral ground-state X~A11 to the Rydberg series converging to the ionic electronic ground-state (2t_1_)^−1^ X~T12 is dipole forbidden. The lowest-lying Rydberg transition (*n* = 4) converging to the ionic electronic first excited-state IE_2_, (7t_2_)^−1^ A~T22, is assigned to the $\left(4s\leftarrow 7t_{2}\right)$ excitation, with the first member at 8.92(9) eV and with a quantum defect *δ* = 1.98. Other works report such a feature at 8.859 eV [11], 8.94 eV [14], 8.853 eV [13] and 8.87 eV [39]. The Rydberg 5s member $\left(5s\leftarrow 7t_{2}\right)$ of the series is at 10.74(9) eV with *δ* = 2.00 (Table 3). The first member of the *np* $\left(np\leftarrow 7t_{2}\right)$ series has an absorption feature at 9.60(4) eV, *δ* = 1.74 $\left(4p\leftarrow 7t_{2}\right)$, while Causley and Russell [11] reported it at 9.611 eV, Ho [14] at 9.67 eV and Russell et al. [13] at 9.596 eV. For the $\left(np^{\prime }\leftarrow 7t_{2}\right)$ series, the assigned *n* = 4 absorption feature is at 10.26(4) eV with a quantum defect *δ* = 1.39 $\left(4p^{\prime }\leftarrow 7t_{2}\right)$. Finally, Table 3 also includes two *nd* $\left(nd\leftarrow 7t_{2}\right)$ and $\left(nd\prime \leftarrow 7t_{2}\right)$ series, where only *n* = 3d and *n* = 3d′ are discernible at 10.61(5) eV, *δ* = 0.13 $\left(3d\leftarrow 7t_{2}\right)$ and 10.74(9) eV, *δ* ≈ 0.00 $\left(3d^{\prime }\leftarrow 7t_{2}\right)$, with the former reported by Ho [14] at 10.63 eV. No further assignments for higher members of the Rydberg series have been performed because those features lie outside the photon energy range investigated.

The Rydberg transitions in the absorption spectrum are accompanied by fine structures, which have been assigned in Table 2. The information on the modes contributing to the spectrum has been obtained from the calculation of the harmonic frequencies of the cationic electronic ground-state in the *C*_1_ point group (Table S4). The major contributions are from the C–Cl stretching/deformation, $v_{6}^{\prime }\left(a\right)$ (0.059 eV, 476.2 cm^−1^), and the asymmetric stretching, $v_{9}^{\prime }\left(a\right)$ (0.115 eV, 929.6 cm^−1^), modes. It is interesting to note that the cationic electronic ground-state is not stable under *T*_d_ symmetry and the molecular framework undergoes a geometry change to lower its symmetry, thus becoming more stable. Such a Jahn–Teller effect for CCl_4_^+^ is also known for other carbon tetrahalide molecules (CX_4_^+^, X = F, Cl, Br) [3], as it is also known for CH_4_^+^ [41]. The electronic excitation of the carbon tetrachloride molecule from the neutral ground-state to the cationic electronic ground-state (geometry, bond lengths in Å and bond angles in (°) are shown in Figure S2) yields a C1–Cl_2_/C1–Cl_5_ bond length decrease by ~4% and an enhancement of ~4% in the C1–Cl_3_/C1–Cl_4_ interatomic distance, while the major difference is noted from the 20% reduction in the Cl_3_–C1–Cl_4_ angle. These changes are in accordance with the C–Cl stretching/deformation, $v_{6}^{\prime }\left(a\right)$, and asymmetric stretching, $v_{9}^{\prime }\left(a\right)$, modes, assigned in Table 2.

### 3.5. Absolute Photoabsorption Cross-Sections

The high-resolution vacuum ultraviolet photoabsorption cross-sections of carbon tetrachloride are reported in the photon energy region 5.0–10.8 eV, with Table 1 listing the major electronic transitions and their values in units of Mb. A previous study of the vacuum ultraviolet photoabsorption has covered the wavelength region 180 to 240 nm (6.888–5.166 eV) [42] and is found to be in reasonable agreement with the present cross-sections (<3% lower). Causley and Russell [11] report, at 56,790 cm^−1^ (7.041 eV), 71,450 cm^−1^ (8.859 eV), 75,130 cm^−1^ (9.315 eV) and 77,520 cm^−1^ (9.611 eV), cross-section values of 3.02, 24.58, 71.74 and 74.53 Mb which are much lower than our values of 10.84, 43.78, 131.48 and 135.60 Mb, respectively. Making a close comparison of earlier results with the present, Ho [14] reports absolute cross-sections which are 13–44% lower from 6.2 to 8.0 eV and 1–14% higher from 9.0 to 10.8 eV than our values. We also note a disagreement on the magnitude of the two strongest absorption bands, with the present values of 140.41 and 144.31 Mb at 9.343 and 9.652 eV, while Ho [14] reports values of 148.8 and 144.6 Mb at 9.4 and 9.6 eV, respectively. The other experimental values, from an optical absorption experiment, report cross-sections at 175 nm (7.085 eV) of 11.8 Mb and at 138.8 nm (8.933 eV) of 54.5 Mb [21], which are 8 and 18% higher than our corresponding results of 10.83 and 44.47 Mb (Figure 1). The absorption maxima of Russell et al. [13] at 56,800 cm^−1^ (7.042 eV) and at 71,400 cm^−1^ (8.852 eV) are 12.09 and 142.74 Mb, compared with the present data of 10.84 and 43.65 Mb. The photoabsorption data of Lee and Suto [12] in shape and magnitude compare quite well with our corresponding results in Figure 1.

## 4. Materials and Methods

The AU-UV beam line of the ASTRID2 synchrotron facility at Aarhus University, Denmark has been used to obtain a high-resolution VUV photoabsorption spectrum of carbon tetrachloride in the energy range from 4.0 eV to 10.8 eV (Figure 1 and Figure 2). The AU-UV beam line is capable of delivering VUV radiation with a resolution better than 0.08 nm [43], which corresponds to 1, 3 and 7 meV at the low extreme, the midpoint, and the high extreme of the photon energy range scanned, respectively. The gas-phase experiments were performed in an absorption gas cell end station, which has been described elsewhere [43,44]. In short, synchrotron radiation passes through a static gas sample of CCl_4_ vapour at room temperature with the transmitted light detected by a photomultiplier tube (PMT). Two MgF_2_ transmission windows placed at each end of the absorption cell set the lower wavelength limit to 115 nm. The gas sample number density in the absorption cell is obtained by recording the absolute pressure of CCl_4_ measured by a capacitance manometer (Chell CDG100D), while the absorption cross-sections were measured in the pressure range 0.01–1.27 mbar to achieve attenuations of 50% or less and hence avoid saturation effects.

The absolute photoabsorption cross-sections values, *σ*, in units of megabarn (1 Mb ≡ 10^−18^ cm^2^), were obtained using the Beer–Lambert attenuation law, $I_{t}=I_{0}e^{\left(-N\sigma l\right)}$, where *I_t_* is the light intensity transmitted through the gas sample, *I*_0_ is that through the evacuated cell, *N* the molecular number density of carbon tetrachloride, and *l* the absorption path length (15.5 cm). ASTRID2 operates in a “top-up” mode allowing the light intensity to be kept quasi-constant, thus compensating for the constant beam decay in the storage ring. The synchrotron beam current is monitored throughout the collection of each spectrum, and background scans (*I*_0_) were recorded with the cell evacuated. Within the wavelength region scanned (115–260 nm), accurate cross-section values are obtained by recording the VUV spectrum in small (5 or 10 nm) sections, allowing an overlap of at least 10 points between the adjoining sections. The methodology was employed to determine photoabsorption cross-sections to an accuracy of ±5%.

The liquid sample used in the VUV photoabsorption measurements was purchased from Merck, with a stated purity of ≥99.95%. The sample was degassed through repeated freeze–pump–thaw cycles before use.

The assignment of the major electronic excitations in the photoabsorption spectrum of CCl_4_ has been performed with the help of quantum chemical calculations, providing important information on its electronic and molecular structures. The vertical excitation energies and oscillator strengths of the major electronically excited-states listed in Table 1 were calculated employing TD-DFT [45,46] with the PBE0 functional [47,48,49,50,51] and the aug-cc-pVDZ basis set [52] as implemented in the GAMESS-US computational package [53]. CCl_4_ belongs to the point group *T*_d_ which is a non-Abelian symmetry group; thus, the calculations were performed using the smallest Abelian sub-group *C*_2v_. A close comparison of the calculated dominant transitions with the corresponding experimental absorption features shows a very good agreement to within ±7%. Within an electronic excitation from the ground-state, the underlying calculation procedure takes the molecular structure at its ground-state equilibrium geometry, and the molecular framework is thus kept rigid within the accessible Franck–Condon region of the transition. However, we have comprehensively investigated the nuclear dynamics along the degenerate deformation mode, $v_{4}^{\prime }\left(t_{2}\right)$, to obtain the vertical excitation energy of the lowest-lying excited-state at 6.156 eV (Figure 1), which is not provided by the calculations (see Section 3.1) in the Born–Oppenheimer approximation. The resulting potential energy curves are plotted in Figure 3. Harmonic frequencies (DFT/PBE0/aug-cc-pVDZ) for the neutral electronic ground-state (Table S3) and the cationic electronic ground-state (Table S4) have also been obtained, although the neutral electronic first excited-state does not show any stable structure due to fast C–Cl bond excision (see Section 3.1).

## 5. Conclusions

In the present investigation, we report the most comprehensive study of the geometry and the electronic structure of carbon tetrachloride lowest-lying states by combining experimental and theoretical methodologies. The high-resolution VUV photoabsorption spectrum in the 5.0–10.8 eV region exhibits features that have been assigned to valence and Rydberg transitions with the aid of Time-Dependent Density Functional Theory calculations on the vertical excitation energies and oscillator strengths. The detailed analysis of the structure on the photoabsorption features has also allowed us to propose assignments for the degenerate stretching, $v_{3}^{\prime }\left(t_{2}\right)$, and degenerate deformation, $v_{4}^{\prime }\left(t_{2}\right),$ modes.

We have obtained, at the TD-DFT/aug-cc-pVDZ level of theory, potential energy curves for the lowest-lying excited singlet states of carbon tetrachloride as a function of the degenerate deformation mode, $v_{4}^{\prime }\left(t_{2}\right)$, mode. The relevant symmetry breaking due to the Jahn–Teller distortion of the electronically excited CCl_4_ molecule is shown here, for the first time, to be responsible for the weak absorption feature at a vertical excitation energy of 6.156 eV. Additional calculations at the same level of theory have also been shown for the singlet excited-states of carbon tetrachloride along the C–Cl stretching coordinate while keeping all the vibrational modes frozen, showing prompt dissociation-yielding Cl species.

## Figures and Tables

**Table 1:** The most representative calculated vertical excitation energies (TD-DFT/PBE0/aug-cc-pVDZ) and oscillator strengths of carbon tetrachloride compared with the present experimental data. Energies in eV. See the text for details.

Carbon Tetrachloride, CCl_4_					E (eV) Expt. ^a^	Cross-Section (Mb)
State (C_2v_)	State (T_d_)	E (eV)	f_L_	Dominant Excitations		
$\tilde{X}$ ^1^A_1_	$\tilde{X}$ ^1^A_1_					
2 ^1^A_1_	1 ^1^T_2_	6.853	0.0202	$\sigma^{*}\left(16a_{1}\right)\leftarrow n_{Cl}/\sigma \left(15a_{1}\right)$ (87%)		
1 ^1^B_2_				$\sigma^{*}\left(16a_{1}\right)\leftarrow n_{Cl}/\sigma \left(8b_{2}\right)$ (87%)	7.06(1)	10.87
1 ^1^B_1_				$\sigma^{*}\left(16a_{1}\right)\leftarrow n_{Cl}/\sigma \left(8b_{1}\right)$ (87%)		
3 ^1^B_2_	3 ^1^T_2_	8.468	0.1254	$\sigma^{*}\left(10b_{2}\right)\leftarrow n_{Cl}/\sigma \left(15a_{1}\right)$$\;(42\%)+\sigma^{*}\left(17a_{1}\right)\leftarrow n_{Cl}/\sigma \left(8b_{2}\right)$ (42%)		
3 ^1^B_1_				$\sigma^{*}\left(10b_{1}\right)\leftarrow n_{Cl}/\sigma \left(15a_{1}\right)$$\;(42\%)+\sigma^{*}\left(17a_{1}\right)\leftarrow n_{Cl}/\sigma \left(8b_{1}\right)$$\;(42\%)+\sigma^{*}\left(10b_{2}\right)\leftarrow n_{Cl}\left(3a_{2}\right)$ (6%)	8.92(9)	44.48
4 ^1^A_1_				$\sigma^{*}\left(10b_{2}\right)\leftarrow n_{Cl}/\sigma \left(8b_{2}\right)$$\;(42\%)+\sigma^{*}\left(10b_{1}\right)\leftarrow n_{Cl}/\sigma \left(8b_{1}\right)$ (42%)		
4 ^1^B_2_	4 ^1^T_2_	9.047	0.0358	$\sigma^{*}\left(10b_{2}\right)\leftarrow n_{Cl}\left(14a_{1}\right)$$\;(20\%)+\sigma^{*}\left(10b_{1}\right)\leftarrow n_{Cl}\left(3a_{2}\right)$$\;(60\%)+4s\left(18a_{1}\right)\leftarrow n_{Cl}/\sigma \left(8b_{2}\right)$ (4%)		
4 ^1^B_1_				$\sigma^{*}\left(10b_{2}\right)\leftarrow n_{Cl}\left(3a_{2}\right)$$\;(60\%)+\sigma^{*}\left(10b_{1}\right)\leftarrow n_{Cl}\left(14a_{1}\right)$$\;(20\%)+4s\left(18a_{1}\right)\leftarrow n_{Cl}/\sigma \left(8b_{1}\right)$ (4%)	9.343	140.41
5 ^1^A_1_				$\sigma^{*}\left(17a_{1}\right)\leftarrow n_{Cl}\left(14a_{1}\right)$$\;(80\%)+4s\left(18a_{1}\right)\leftarrow n_{Cl}/\sigma \left(15a_{1}\right)$ (4%)		
5 ^1^B_1_	5 ^1^T_2_	9.339	0.2174	$4s\left(18a_{1}\right)\leftarrow n_{Cl}/\sigma \left(8b_{1}\right)$$\;(54\%)+4p^{\prime }\left(11b_{2}\right)\leftarrow n_{Cl}\left(4a_{2}\right)$$\;(16\%)+\sigma^{*}\left(10b_{2}\right)\leftarrow n_{Cl}\left(3a_{2}\right)$ (6%)		
5 ^1^B_2_				$4s\left(18a_{1}\right)\leftarrow n_{Cl}/\sigma \left(8b_{2}\right)$$\;(54\%)+4p\left(11b_{1}\right)\leftarrow n_{Cl}\left(4a_{2}\right)$$\;(16\%)+\sigma^{*}\left(10b_{1}\right)\leftarrow n_{Cl}\left(3a_{2}\right)$ (6%)	9.652	144.31
6 ^1^A_1_				$4s\left(18a_{1}\right)\leftarrow n_{Cl}/\sigma \left(15a_{1}\right)$$\;(54\%)+4p\left(11b_{1}\right)\leftarrow n_{Cl}\left(9b_{1}\right)$$\;(16\%)+4p^{\prime }\left(11b_{2}\right)\leftarrow n_{Cl}\left(9b_{2}\right)$$\;(16\%)+\sigma^{*}\left(17a_{1}\right)\leftarrow n_{Cl}\left(14a_{1}\right)$ (8%)		
7 ^1^A_1_	6 ^1^T_2_	9.584	0.0157	$4s\left(18a_{1}\right)\leftarrow n_{Cl}/\sigma \left(15a_{1}\right)$$\;(41\%)+4p\left(11b_{1}\right)\leftarrow n_{Cl}\left(9b_{1}\right)$$\;(28\%)+4p^{\prime }\left(11b_{2}\right)\leftarrow n_{Cl}\left(9b_{2}\right)$ (28%)		
6 ^1^B_2_				$4s\left(18a_{1}\right)\leftarrow n_{Cl}/\sigma \left(8b_{2}\right)$$\;(41\%)+4p\left(11b_{1}\right)\leftarrow n_{Cl}\left(4a_{2}\right)$ (28%)	10.26(4)	22.16
6 ^1^B_1_				$4s\left(18a_{1}\right)\leftarrow n_{Cl}/\sigma \left(8b_{1}\right)$$\;(41\%)+4p^{\prime }\left(11b_{2}\right)\leftarrow n_{Cl}\left(4a_{2}\right)$ (28%)		
8 ^1^A_1_	7 ^1^T_2_	10.344	0.0155	$4p\left(11b_{1}\right)\leftarrow n_{Cl}/\sigma \left(8b_{1}\right)$$\;(41\%)+4p^{\prime }\left(11b_{2}\right)\leftarrow n_{Cl}/\sigma \left(8b_{2}\right)$$\;(41\%)+3d\left(5a_{2}\right)\leftarrow n_{Cl}\left(4a_{2}\right)$ (14%)		
7 ^1^B_1_				$4p\left(11b_{1}\right)\leftarrow n_{Cl}/\sigma \left(15a_{1}\right)$$\;(41\%)+3d^{\prime }\left(20a_{1}\right)\leftarrow n_{Cl}\left(9b_{1}\right)$ (11%)		
7 ^1^B_2_				$4p^{\prime }\left(11b_{2}\right)\leftarrow n_{Cl}/\sigma \left(15a_{1}\right)$$\;(41\%)+3d^{\prime }\left(20a_{1}\right)\leftarrow n_{Cl}\left(9b_{2}\right)$ (11%)		

^a^ The last decimal of the energy value is given in brackets for these less-resolved features.

**Table 2:** Proposed vibrational assignments of carbon tetrachloride valence and Rydberg series converging to (7t2)−1 A~T22 in the photon energy range 8.5−10.0 eV ^a^. Energies in eV. See the text for details.

		*T*_d_		*C*_1_
Assignment	Energy	$\Delta E\;\left(\nu_{3}^{\prime }\right)$	$\Delta E\;\left(\nu_{4}^{\prime }\right)$	Assignment
σ*←σ+σ*←nCl/σ, 3T21←X~A11				
$0_{0}^{0}$	8.52(1)(s,w)	–	–	–
$3_{0}^{1}$	8.63(4)(w)	0.113	–	–
$3_{0}^{2}$	8.73(4)(w)	0.100	–	–
$3_{0}^{3}$	8.81(8)(s)	0.084	–	–
$3_{0}^{4}/4s{\left(7t_{2}\right)}^{-1}$	8.92(9)(b)	0.111	–	$9_{0}^{0}$
$3_{0}^{5}$	9.03(7)(s,w)	0.108	–	$9_{0}^{1}$
$3_{0}^{6}$	9.14(7)(s,b)	0.110	–	$9_{0}^{2}$
4s←nCl/σ+σ*←nCl, 4T21←X~A11				
$0_{0}^{0}$	9.14(7)(s,b)	–	–	–
$4_{0}^{1}$	9.19(1)(s)	–	0.044	–
$4_{0}^{2}$	9.22(5)(s,w)	–	0.034	–
$3_{0}^{1}$	9.24(2)(s,w)	0.095	–	–
$4_{0}^{3}$	9.26(3)(s,w)	–	0.038	–
$4_{0}^{4}$	9.30(1)(s,w)	–	0.038	–
$3_{0}^{2}/4_{0}^{5}$	9.343	0.101	0.042	–
$4_{0}^{6}$	9.37(9)(b)	–	0.036	–
$4_{0}^{7}$	9.41(1)(s,w)	–	0.032	–
4s←nCl/σ+σ*←nCl/σ, 5T21←X~A11				
$0_{0}^{0}$	9.55(2)(s,w)	–	–	–
$4_{0}^{1}/4p{\left(7t_{2}\right)}^{-1}$	9.60(4)(b)	–	0.052	$9_{0}^{0}$
$3_{0}^{1}$	9.652	0.100	–	$9_{0}^{1}$
$3_{0}^{1}4_{0}^{1}$	9.70(1)(b)	0.101	0.052	$9_{0}^{1}6_{0}^{1}$
$3_{0}^{2}$	9.75(1)(b,w)	0.099	–	$9_{0}^{2}$
$3_{0}^{2}4_{0}^{1}$	9.80(5)(s,w)	0.104	0.054	$9_{0}^{2}6_{0}^{1}$
$3_{0}^{3}$	9.84(4)(s,w)	0.093	–	$9_{0}^{3}$
$3_{0}^{4}$	9.94(7)(s)	0.103	–	$9_{0}^{4}$
	$\bar{\Delta E}$	0.102	0.042	

^a^ (s) shoulder structure; (w) weak feature; (b) broad structure (the last decimal of the energy value is given in brackets for these less-resolved features).

**Table 3:** Energy values (eV), quantum defects (*δ*) and assignments of the Rydberg series converging to (7t_2_)^−1^ A~T22 of carbon tetrachloride. ^a^ See the text for details.

*E*_n_	*δ*	Assignment
$({\mathrm{IE}}_{2}{)}_{\mathrm{ad}}\;=\;12.27\;\mathrm{eV}\;{\left(7t_{2}\right)}^{-1}$		
(*ns* ← 7*t*_2_)		
8.92(9)(b)	1.98	4s
10.74(9)(s,w)	2.00	5s
(*np* ← 7*t*_2_)		
9.60(4)(s)	1.74	4p
(*np’* ← 7*t*_2_)		
10.26(4)(s,w)	1.39	4p’
(*nd* ← 7*t*_2_)		
10.61(5)(s)	0.13	3d
(*nd’* ← 7*t*_2_)		
10.74(9)(s,w)	0.00	3d’

^a^ (b) broad structure; (s) shoulder structure; (w) weak feature (the last decimal of the energy value is given in brackets for these less-resolved features).

## Data Availability

Data presented in this publication are available upon request to the authors.

## Supplementary Materials

The following supporting information can be downloaded at https://www.mdpi.com/article/10.3390/molecules29235619/s1, Figure S1: Neutral ground-state geometry of carbon tetrachloride optimised at the DFT/PBE0/aug-cc-pVDZ level in the *T*_d_ point group. Bond lengths are in Å and bond angles in (°). Cartesian coordinates in Å and electronic configuration of $\tilde{X}$ ^1^A_1_ state; Figure S2: Cation ground-state geometry of carbon tetrachloride optimised at the DFT/PBE0/aug-cc-pVDZ level in the *C*_1_ point group. Bond lengths are in Å and bond angles in (°). Cartesian coordinates in Å; Figure S3: Representation of a selection of carbon tetrachloride molecular orbitals at the DFT/PBE0/aug-cc-pVDZ level according to the *C*_2v_ point group; Figure S4. Representation of a selection of carbon tetrachloride molecular orbitals at the DFT/PBE0/aug-cc-pVTZ level according to the *C*_2v_ point group; Figure S5: PECs for the singlet excited-states of carbon tetrachloride along the C1–Cl3 coordinate, while keeping all coordinates of other atoms frozen. The calculations were performed at the TD-DFT/PBE0/aug-cc-pVDZ level of theory in the *C*_1_ symmetry group; Figure S6: PECs for the singlet excited-states of carbon tetrachloride along the Cl_2_–C1–Cl_3_ coordinate, while keeping while keeping all coordinates of other atoms frozen. The calculations were performed at the TD-DFT/PBE0/aug-cc-pVDZ level of theory in the *C*_1_ symmetry group; Figure S7: Neutral first excited-state geometry of carbon tetrachloride optimised at the DFT/PBE0/aug-cc-pVDZ level in the *C*_1_ point group. Bond lengths are in Å and bond angles in (°). Cartesian coordinates in Å; Table S1: The calculated vertical excitation energies (TD-DFT/PBE0/aug-cc-pVDZ) and oscillator strengths of carbon tetrachloride. Energies in eV; Table S2. The calculated vertical excitation energies (TD-DFT/PBE0/aug-cc-pVTZ) and oscillator strengths of carbon tetrachloride. Energies in eV; Table S3: Harmonic frequencies from the DFT/PBE0/aug-cc-pVDZ level for carbon tetrachloride neutral electronic ground-state, in the *C*_2v_ (and *T*_d_) point group, compared with experimental data; Table S4: Harmonic frequencies from the DFT/PBE0/aug-cc-pVDZ level for carbon tetrachloride cationic electronic ground-state in the *C*_1_ point group. Citation of Ref. [54].
