# Supplementary material for: Symmetry Breaking in the Lowest-Lying Excited-State of CCl4: Valence Shell Spectroscopy in the 5.0–10.8 eV Photon Energy Range
Source: Molecules. 2024 Nov 27;29(23):5619. doi: 10.3390/molecules29235619 (PMC11643474; doi:10.3390/molecules29235619)
Supplement: Supplementary file 1 [file molecules-29-05619-s001.zip › molecules-3332649-supplementary.pdf]

Supplementary Material

Symmetry Breaking in the Lowest-Lying Excited-State of CCl_4: Valence Shell Spectroscopy in the 5.0–10.8 eV Photon Energy Range

Luiz V. S. Dalagnol [1], Sarvesh Kumar [2,3], Alessandra S. Barbosa [1], Umma S. Akther [2], Nykola C. Jones [4],
Søren V. Hoffmann [4], Márcio H. F. Bettega [1,*] and Paulo Limão-Vieira [2,*]

[1] Departamento de Física, Universidade Federal do Paraná, Caixa Postal 19044, Curitiba 81531-980, Paraná, Brazil; lvsd15@fisica.ufpr.br (L.V.S.D.); alessandra@fisica.ufpr.br (A.S.B.)
[2] Atomic and Molecular Collisions Laboratory, CEFITEC-Centre of Physics and Technological Research, Department of Physics, NOVA School of Science and Technology, Universidade NOVA de Lisboa, 2829-516 Caparica, Portugal; s.kumar@campus.fct.unl.pt (S.K.); u.akther@campus.fct.unl.pt (U.S.A.)
[3] Chemical Sciences Division, Lawrence Berkeley National Laboratory, One Cyclotron Road, Berkeley 94720, CA, USA; skumar2@lbl.gov
[4] ISA, Department of Physics and Astronomy, Aarhus University, Ny Munkegade 120, DK-8000 Aarhus C, Denmark; nykj@phys.au.dk (N.C.J.); vronning@phys.au.dk (S.V.H.)
* Correspondence: bettega@fisica.ufpr.br (M.H.F.B.); plimaovieira@fct.unl.pt (P.L.-V.); Tel.: +55-41-3361-3002 (M.H.F.B.); +351-21-294-78-59 (P.L.-V.)

Figure captions

Figure S1. Neutral ground-state geometry of carbon tetrachloride optimised at the DFT/PBE0/aug-cc-pVDZ level in the T_d point group. Bond lengths are in Å and bond angles in (°). Cartesian coordinates in Å and electronic configuration of $\tilde{X}$ $^1A_1$ state.

Figure S2. Cation ground-state geometry of carbon tetrachloride optimised at the DFT/PBE0/aug-cc-pVDZ level in the C_1 point group. Bond lengths are in Å and bond angles in (°). Cartesian coordinates in Å.

Figure S3. Representation of a selection of carbon tetrachloride molecular orbitals at the DFT/PBE0/aug-cc-pVDZ level according to the C_{2v} point group.

Figure S4. Representation of a selection of carbon tetrachloride molecular orbitals at the DFT/PBE0/aug-cc-pVTZ level according to the C_{2v} point group.

Figure S5. PECs for the singlet excited states of carbon tetrachloride along the C1–Cl3 coordinate, while keeping all coordinates of other atoms frozen. The calculations were performed at the TD-DFT/PBE0/aug-cc-pVDZ level of theory in the C_1 symmetry group. See text for details.

Figure S6. PECs for the singlet excited states of carbon tetrachloride along the Cl2–C1–Cl3 coordinate, while keeping while keeping all coordinates of other atoms frozen. The calculations were performed at the TD-DFT/PBE0/aug-cc-pVDZ level of theory in the C_1 symmetry group. See text for details.

Figure S7. Neutral first excited-state geometry of carbon tetrachloride optimised at the DFT/PBE0/aug-cc-pVDZ level in the C_1 point group. Bond lengths are in Å and bond angles in (°). Cartesian coordinates in Å.

Table caption

Table S1. The calculated vertical excitation energies (TD-DFT/PBE0/aug-cc-pVDZ) and oscillator strengths of carbon tetrachloride. Energies in eV. See text for details

Table S2. The calculated vertical excitation energies (TD-DFT/PBE0/aug-cc-pVTZ) and oscillator strengths of carbon tetrachloride. Energies in eV.

Table S3. Harmonic frequencies from the DFT/PBE0/aug-cc-pVDZ level for carbon tetrachloride neutral electronic ground-state, in the C_{2v} (and T_d) point group, compared with experimental data.

Table S4. Harmonic frequencies from the DFT/PBE0/aug-cc-pVDZ level for carbon tetrachloride cationic electronic ground-state in the C_1 point group.

Figure S1. Neutral ground-state geometry of carbon tetrachloride optimised at the DFT/PBE0/aug-cc-pVDZ level in the T_d point group. Bond lengths are in Å and bond angles in (°). Cartesian coordinates in Å and electronic configuration of $\tilde{X}$ $^1A_1$ state.

Cartesian coordinates of all atoms			
Atom	*x* (Å)	*y* (Å)	*z* (Å)
C1	0.00000000	0.00000000	0.00000000
Cl2	-1.44303679	0.00000000	-1.02038109
Cl3	1.44303679	0.00000000	-1.02038109
Cl4	0.00000000	-1.44303679	1.02038109
Cl5	0.00000000	1.44303679	1.02038109

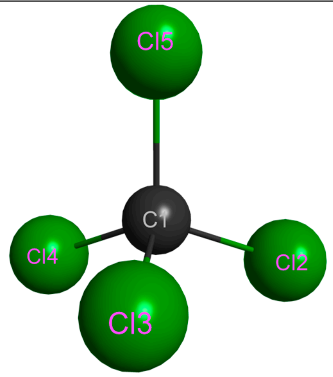

	bond length (Å)		angle (°)
C1 – Cl2	1.767	Cl5 – C1 – Cl2	109.47
C1 – Cl3	1.767	Cl2 – C1 – Cl3	109.47
C1 – Cl4	1.767	Cl3 – C1 – Cl4	109.47
C1 – Cl5	1.767	Cl4 – C1– Cl5	109.47

Electronic configuration of $\tilde{X}$ $^1A_1$ state in C_{2v}:

core orbitals: $(1b_2)^2$ $(1a_1)^2$ $(1b_1)^2$ $(2a_1)^2$ $(3a_1)^2$ $(2b_2)^2$ $(4a_1)^2$ $(2b_1)^2$ $(5a_1)^2$ $(3b_2)^2$ $(6a_1)^2$ $(3b_1)^2$ $(7a_1)^2$ $(4b_2)^2$ $(1a_2)^2$ $(4b_1)^2$ $(8a_1)^2$ $(2a_2)^2$ $(5b_1)^2$ $(9a_1)^2$ $(5b_2)^2$

valence orbitals: $(10a_1)^2$ $(6b_2)^2$ $(11a_1)^2$ $(6b_1)^2$ $(12a_1)^2$ $(13a_1)^2$ $(7b_1)^2$ $(7b_2)^2$ $(14a_1)^2$ $(3a_2)^2$ $(15a_1)^2$ $(8b_1)^2$ $(8b_2)^2$ $(9b_2)^2$ $(4a_2)^2$ $(9b_1)^2$

Unoccupied orbitals: $(16a_1)$ $(10b_2)$ $(10b_1)$ $(17a_1)$ $(18a_1)$ $(11b_1)$ $(11b_2)$ $(19a_1)$ $(5a_2)$ $(20a_1)$

Electronic configuration of $\tilde{X}$ $^1A_1$ state in T_d:

core orbitals: $(1t_2)^6$ $(1a_1)^2$ $(2a_1)^2$ $(2t_2)^6$ $(3a_1)^2$ $(3t_2)^6$ $(4a_1)^2$ $(1t_1)^6$ $(1e)^4$ $(4t_2)^6$

valence orbitals: $(5a_1)^2$ $(5t_2)^6$ $(6a_1)^2$ $(6t_2)^6$ $(2e)^4$ $(7t_2)^6$ $(2t_1)^6$

Unoccupied orbitals: $(7a_1)$ $(8t_2)$ $(8a_1)$ $(9t_2)$ $(3e)$

Figure S2. Cation ground-state geometry of carbon tetrachloride optimised at the DFT/PBE0/aug-cc-pVDZ level in the C_1 point group. Bond lengths are in Å and bond angles in (°). Cartesian coordinates in Å.

Cartesian coordinates of all atoms			
Atom	*x* (Å)	*y* (Å)	*z* (Å)
C1	0.08452000	0.12423600	-0.08961800
Cl2	1.46664906	-0.28838399	-0.99075502
Cl3	-0.23149399	-1.03702199	1.29071999
Cl4	-1.46327198	-0.49961901	-0.84489000
Cl5	-0.02775500	1.75362206	0.38538200

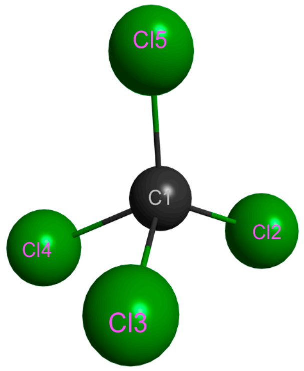

	bond length (Å)		angle (°)
C1 – Cl2	1.701	C1–Cl2–Cl3	112.69
C1 – Cl3	1.831	Cl3–C1–Cl4	87.08
C1 – Cl4	1.832	Cl2–C1–Cl5	115.72
C1 – Cl5	1.701	Cl4–C1–Cl5	115.72

Figure S3. Representation of a selection of carbon tetrachloride molecular orbitals at the DFT/PBE0/aug-cc-pVDZ level according to the C_{2v} point group.

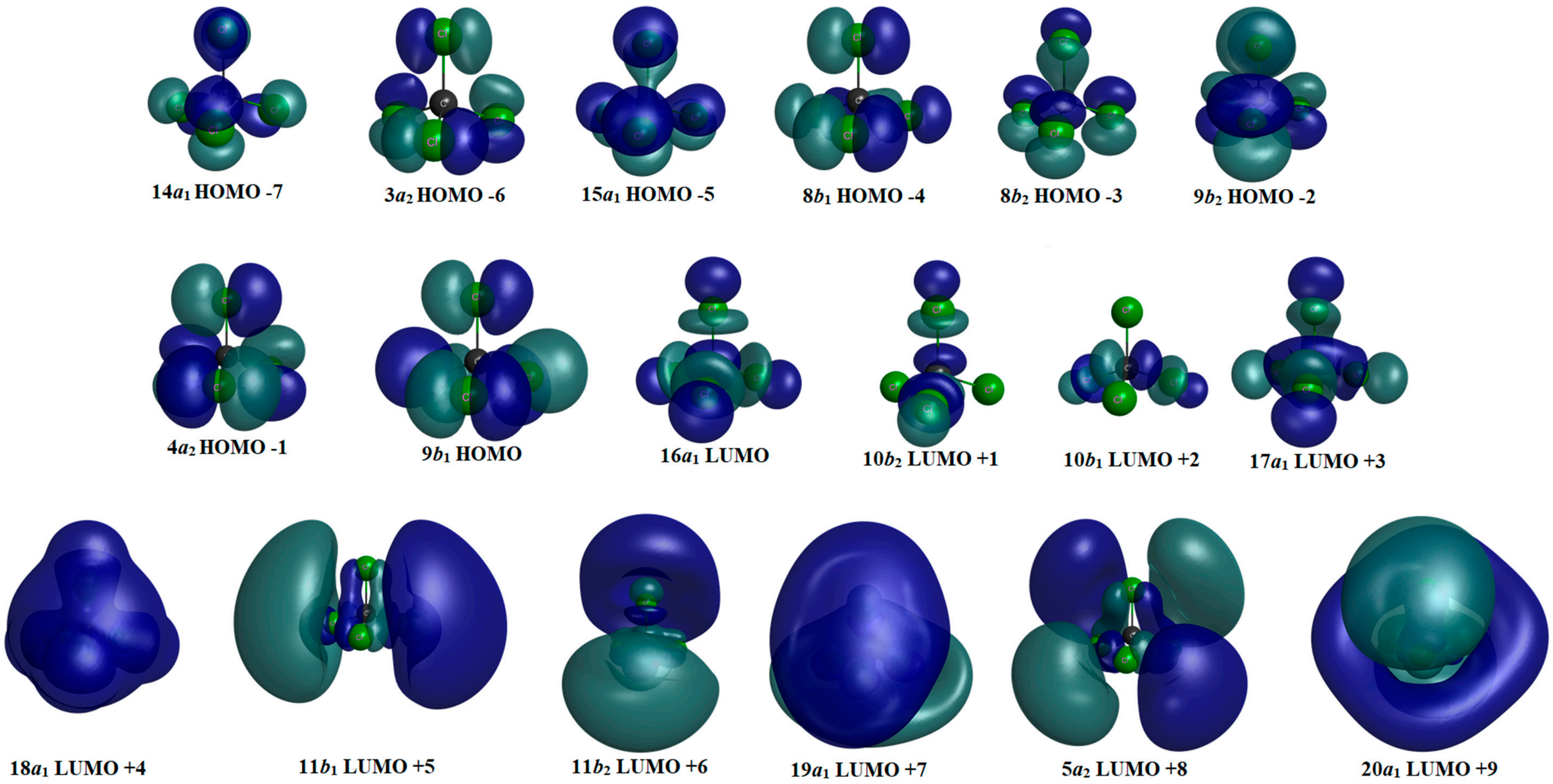

Figure S4. Representation of a selection of carbon tetrachloride molecular orbitals at the DFT/PBE0/aug-cc-pVTZ level according to the C_{2v} point group.

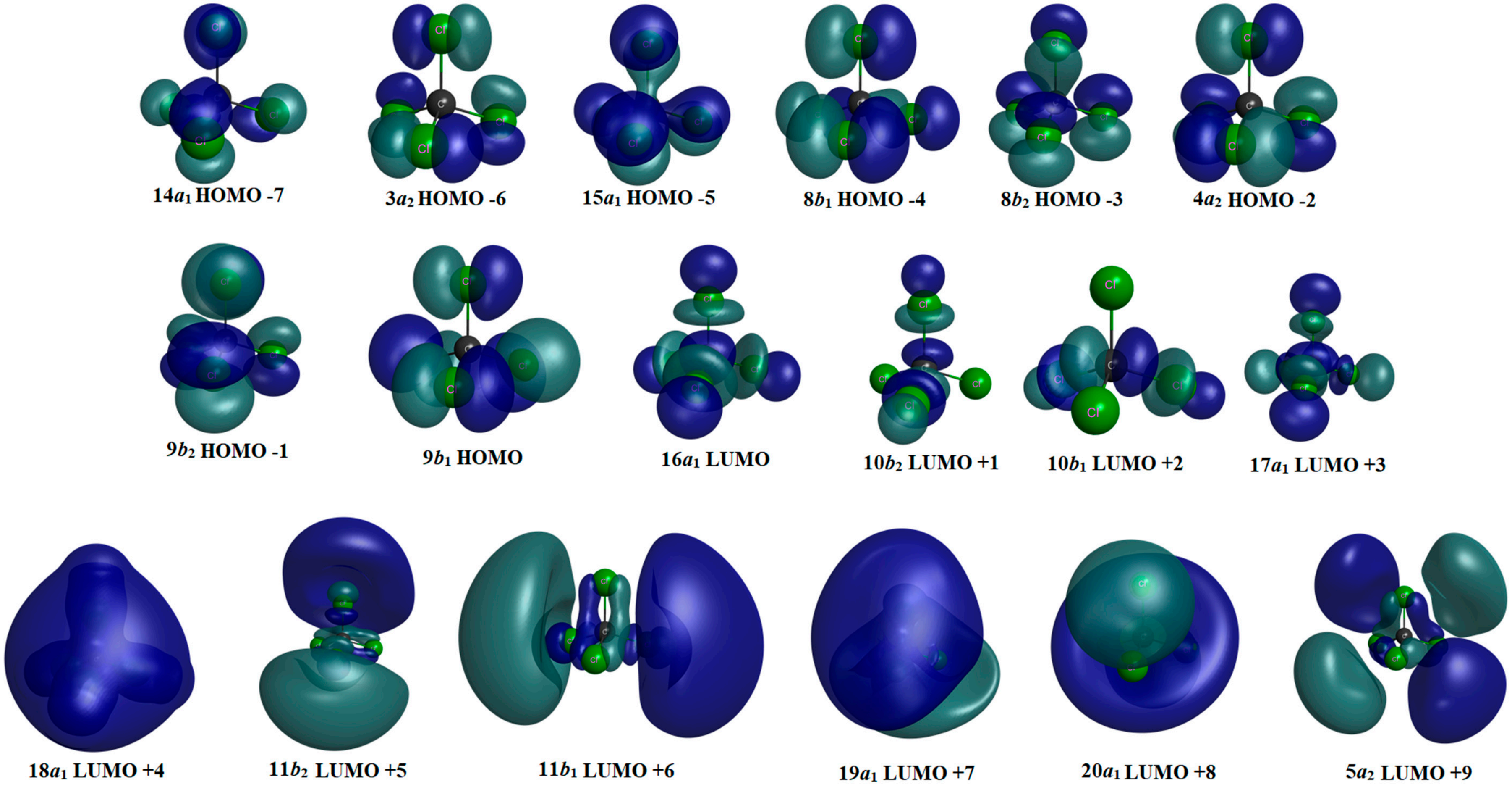

Figure S5. PECs for the singlet excited states of carbon tetrachloride along the C1–Cl3 coordinate, while keeping all coordinates of other atoms frozen. The calculations were performed at the TD-DFT/PBE0/aug-cc-pVDZ level of theory in the C_1 symmetry group. See text for details.

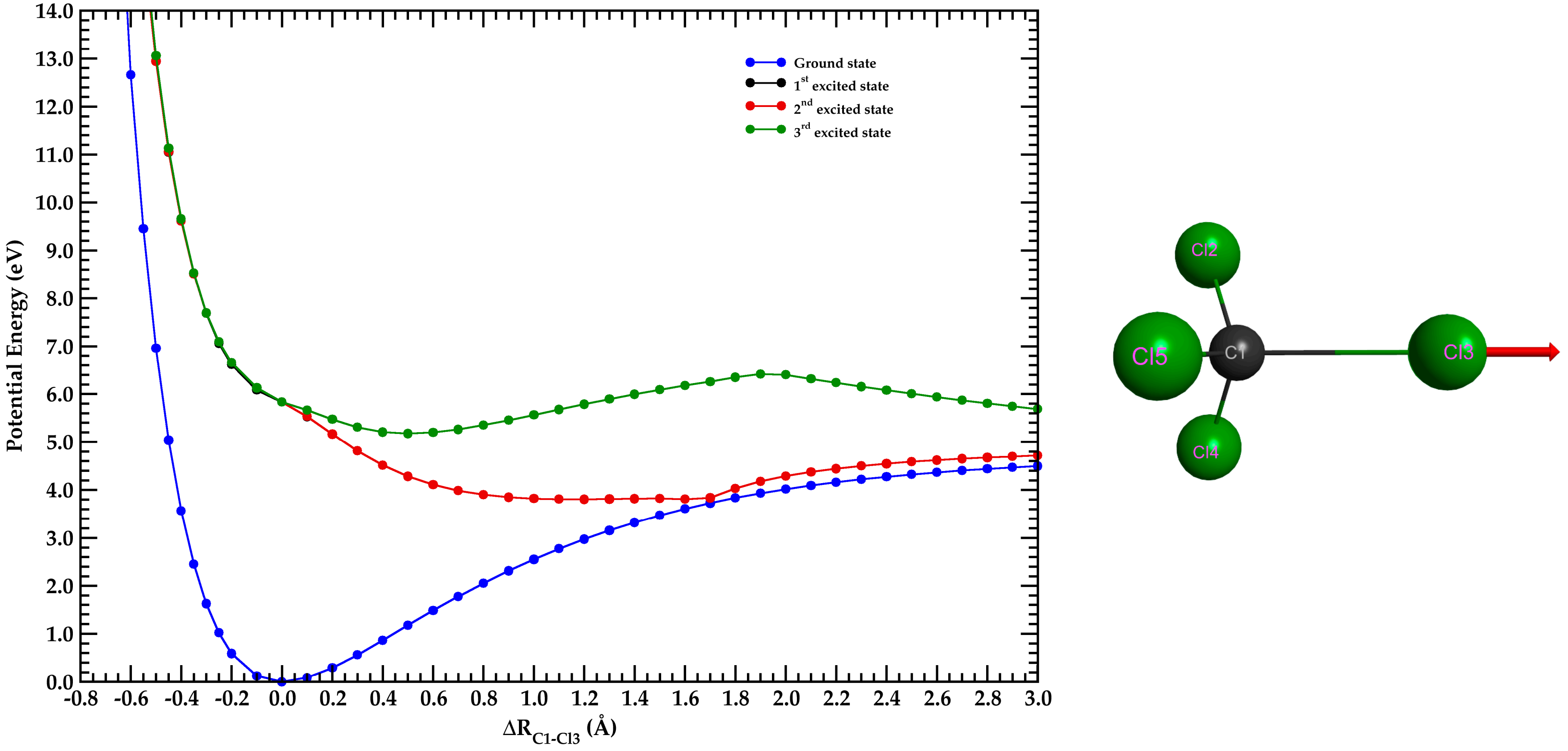

Figure S6. PECs for the singlet excited states of carbon tetrachloride along the Cl2–C1–Cl3 coordinate, while keeping all coordinates of other atoms frozen. The calculations were performed at the TD-DFT/PBE0/aug-cc-pVDZ level of theory in the C_1 symmetry group. See text for details.

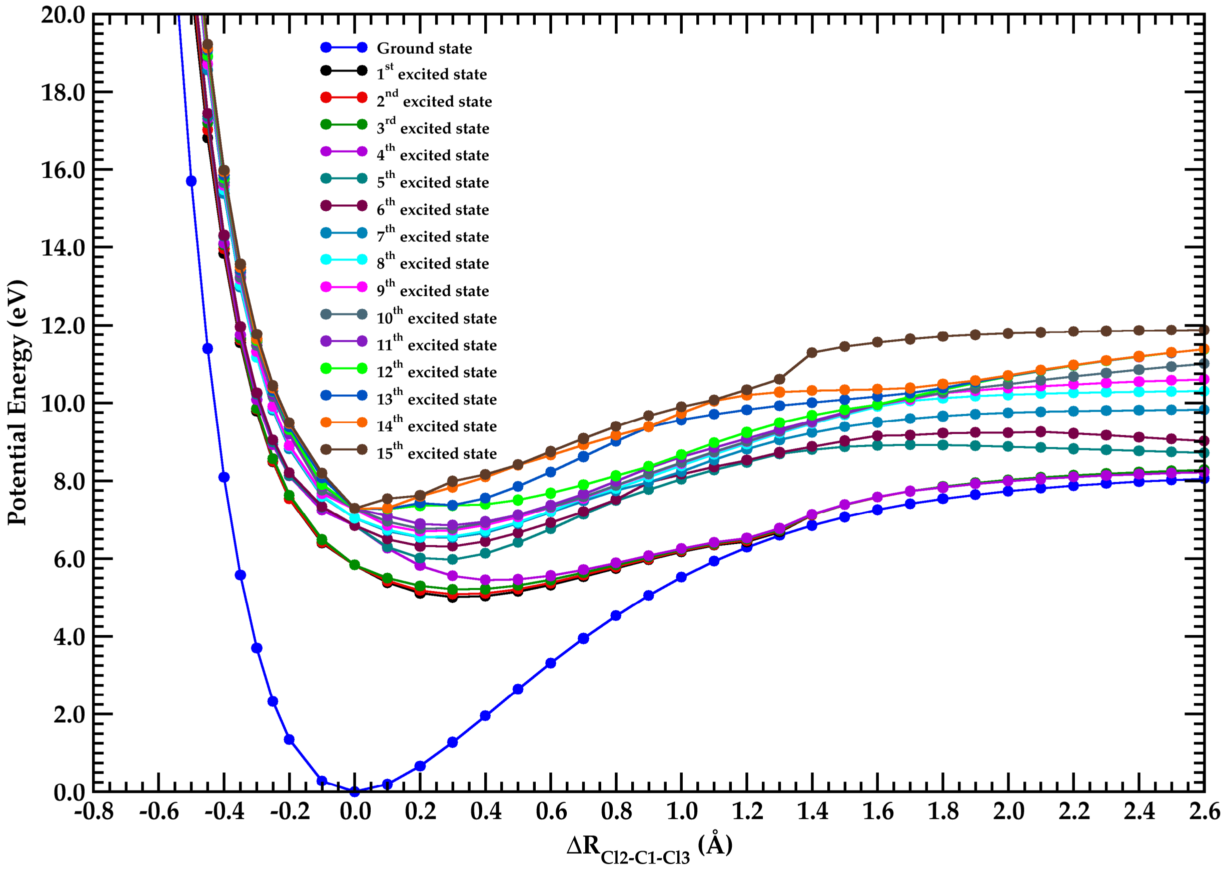

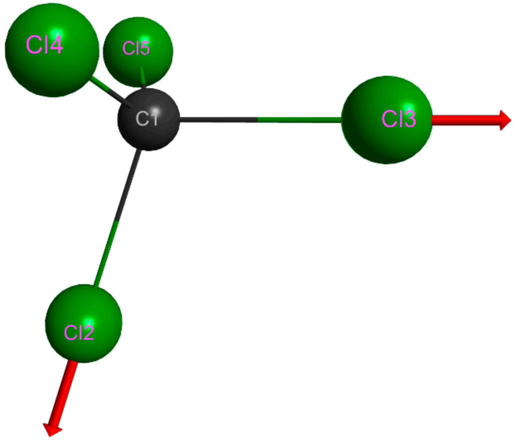

Figure S7. Neutral first excited-state geometry of carbon tetrachloride optimised at the DFT/PBE0/aug-cc-pVDZ level in the C_1 point group. Bond lengths are in Å and bond angles in (°). Cartesian coordinates in Å.

Cartesian coordinates of all atoms			
Atom	*x* (Å)	*y* (Å)	*z* (Å)
C1	0.49749199	0.00010600	0.07971400
Cl2	-1.78199995	0.00034200	1.41105199
Cl3	1.32831001	1.41635895	-0.15886900
Cl4	-1.24447596	-0.00061500	-1.17409098
Cl5	1.32858896	-1.41613305	-0.15783000

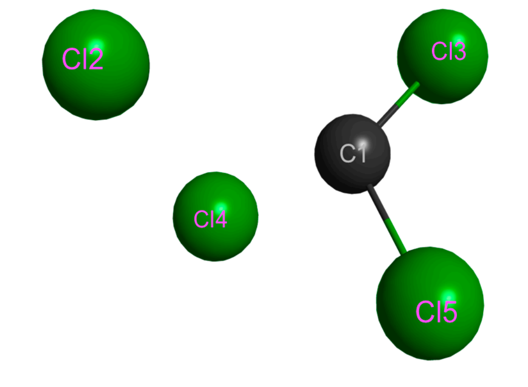

	bond length (Å)		angle (°)
C1 … Cl2*	2.640	Cl5–C1–Cl3	66.03
C1 – Cl3	1.659		
C1 … Cl4*	2.146		
C1 – Cl5	1.659		

* there is no bond length but rather the mutual distance between atoms

Table S1. The calculated vertical excitation energies (TD-DFT/PBE0/aug-cc-pVDZ) and oscillator strengths of carbon tetrachloride. Energies in eV. See text for details.

carbon tetrachloride, CCl_4				
State (C_{2v})	State (T_d)	E (eV)	f_L	Dominant excitations
$^1A_1$	$^1T_2$	6.853378218	0.020168	H-5->LUMO (87%), H-3->L+1 (2%), H-2->L+1 (4%), H-4->L+2 (2%), HOMO->L+2 (4%)
$^1B_2$		6.853403217	0.020178	H-3->LUMO (87%), H-5->L+1 (2%), H-1->L+2 (4%), H-3->L+3 (2%), H-2->L+3 (4%)
$^1B_1$		6.853406217	0.020178	H-4->LUMO (87%), H-1->L+1 (4%), H-5->L+2 (2%), H-4->L+3 (2%), HOMO->L+3 (4%)
$^1A_1$	$^1T_2$	7.291198411	0.012933	H-5->LUMO (10%), H-2->L+1 (43%), HOMO->L+2 (43%)
$^1B_1$		7.291231410	0.012926	H-4->LUMO (10%), H-1->L+1 (43%), HOMO->L+3 (44%)
$^1B_2$		7.291232410	0.012926	H-3->LUMO (10%), H-1->L+2 (43%), H-2->L+3 (44%)
$^1B_2$	$^1T_2$	8.467940614	0.125445	H-5->L+1 (42%), H-3->L+3 (42%), H-7->L+1 (2%), H-6->L+2 (6%)
$^1B_1$		8.467950613	0.125469	H-5->L+2 (42%), H-4->L+3 (42%), H-6->L+1 (6%), H-7->L+2 (2%)
$^1A_1$		8.467980612	0.125451	H-3->L+1 (42%), H-4->L+2 (42%), H-7->L+3 (8%)
$^1B_2$	$^1T_2$	9.046820036	0.035784	H-7->L+1 (20%), H-6->L+2 (60%), H-5->L+1 (2%), H-3->L+3 (2%), H-3->L+4 (4%), H-1->L+5 (4%), H-2->L+7 (4%)
$^1B_1$		9.046825036	0.035798	H-6->L+1 (60%), H-7->L+2 (20%), H-5->L+2 (2%), H-4->L+3 (2%), H-4->L+4 (4%), H-1->L+6 (4%), HOMO->L+7 (4%)
$^1A_1$		9.046895033	0.035801	H-7->L+3 (80%), H-3->L+1 (2%), H-4->L+2 (2%), H-5->L+4 (4%), HOMO->L+5 (4%), H-2->L+6 (4%)
$^1B_1$	$^1T_2$	9.339278145	0.217044	H-4->L+4 (54%), H-1->L+6 (16%), HOMO->L+7 (16%), H-6->L+1 (6%), H-7->L+2 (2%)
$^1B_2$		9.339292144	0.217086	H-3->L+4 (54%), H-1->L+5 (16%), H-2->L+7 (16%), H-7->L+1 (2%), H-6->L+2 (6%)
$^1A_1$		9.339372142	0.216984	H-5->L+4 (54%), HOMO->L+5 (16%), H-2->L+6 (16%), H-7->L+3 (8%)
$^1A_1$	$^1T_2$	9.583768876	0.015665	H-5->L+4 (41%), HOMO->L+5 (28%), H-2->L+6 (28%)
$^1B_2$		9.583771876	0.015636	H-3->L+4 (41%), H-1->L+5 (28%), H-2->L+7 (28%)
$^1B_1$		9.583779876	0.015629	H-4->L+4 (41%), H-1->L+6 (28%), HOMO->L+7 (28%)

$^1A_1$		10.34418516	0.015489	H-4->L+5 (41%), H-3->L+6 (41%), H-1->L+8 (14%)
$^1B_1$	$^1T_2$	10.34420916	0.015496	H-5->L+5 (41%), H-4->L+7 (41%), HOMO->L+9 (11%), H-2->L+8 (4%)
$^1B_2$		10.34421216	0.015488	H-5->L+6 (41%), H-3->L+7 (41%), H-2->L+9 (11%), HOMO->L+8 (4%)
$^1B_1$		10.66015747	0.002522	H-2->L+8 (20%), HOMO->L+9 (60%), H-5->L+5 (6%), H-4->L+7 (7%)
$^1B_2$	$^1T_2$	10.66015947	0.002521	HOMO->L+8 (20%), H-2->L+9 (60%), H-5->L+6 (6%), H-3->L+7 (7%)
$^1A_1$		10.66027547	0.002537	H-1->L+8 (81%), H-4->L+5 (6%), H-3->L+6 (6%), H-7->L+7 (2%)
$^1A_1$		10.88228196	0.068210	H-10->LUMO (14%), H-7->L+7 (10%), H-2->L+11 (34%), HOMO->L+12 (34%), H-1->L+8 (2%)
$^1B_2$	$^1T_2$	10.88231096	0.068248	H-8->LUMO (14%), H-2->L+10 (34%), H-1->L+12 (34%), H-6->L+5 (7%), H-7->L+6 (2%)
$^1B_1$		10.88231196	0.068246	H-9->LUMO (14%), HOMO->L+10 (34%), H-1->L+11 (34%), H-7->L+5 (2%), H-6->L+6 (7%)
$^1B_1$		11.18665067	0.012299	H-7->L+5 (18%), H-6->L+6 (58%), H-9->LUMO (2%), H-3->L+8 (4%), HOMO->L+10 (5%), H-1->L+11 (5%)
$^1B_2$	$^1T_2$	11.18665167	0.012298	H-6->L+5 (58%), H-7->L+6 (18%), H-8->LUMO (2%), H-4->L+8 (4%), H-2->L+10 (5%), H-1->L+12 (5%)
$^1A_1$		11.18718465	0.012266	H-7->L+7 (76%), H-10->LUMO (2%), H-5->L+9 (6%), H-2->L+11 (5%), HOMO->L+12 (5%)

Table S2. The calculated vertical excitation energies (TD-DFT/PBE0/aug-cc-pVTZ) and oscillator strengths of carbon tetrachloride. Energies in eV.

carbon tetrachloride, CCl_4				
State (C_{2v})	State (T_d)	E (eV)	f_L	Dominant excitations
$^1A_1$	$^1T_2$	6.888461594	0.019739	H-5->LUMO (85%), H-3->L+1 (2%), H-1->L+1 (5%), H-4->L+2 (2%), HOMO->L+2 (5%)
$^1B_2$		6.888495593	0.019752	H-3->LUMO (85%), H-5->L+1 (2%), H-2->L+2 (5%), H-3->L+3 (2%), H-1->L+3 (5%)
$^1B_1$		6.888498593	0.019753	H-4->LUMO (85%), H-2->L+1 (5%), H-5->L+2 (2%), H-4->L+3 (2%), HOMO->L+3 (5%)
$^1A_1$	$^1T_2$	7.307740796	0.011174	H-5->LUMO (12%), H-1->L+1 (42%), HOMO->L+2 (42%)
$^1B_1$		7.307789795	0.011161	H-4->LUMO (12%), H-2->L+1 (43%), HOMO->L+3 (42%)
$^1B_2$		7.307791795	0.011161	H-3->LUMO (12%), H-2->L+2 (43%), H-1->L+3 (42%)
$^1B_2$	$^1T_2$	8.457211193	0.119598	H-5->L+1 (42%), H-3->L+3 (42%)
$^1B_1$		8.457223192	0.119627	H-5->L+2 (42%), H-4->L+3 (42%)
$^1A_1$		8.457282191	0.119588	H-3->L+1 (42%), H-4->L+2 (42%)
$^1A_1$	$^1T_2$	9.022852625	0.001550	H-7->L+3 (58%), H-5->L+4 (12%), H-1->L+5 (14%), HOMO->L+6 (14%)
$^1B_2$		9.022919623	0.001540	H-7->L+1 (14%), H-6->L+2 (43%), H-3->L+4 (12%), H-2->L+6 (14%), H-1->L+7 (14%)
$^1B_1$		9.022925623	0.001542	H-6->L+1 (43%), H-7->L+2 (14%), H-4->L+4 (12%), H-2->L+5 (14%), HOMO->L+7 (14%)
$^1A_1$	$^1T_2$	9.211787759	0.230284	H-7->L+3 (31%), H-5->L+4 (31%), H-1->L+5 (14%), HOMO->L+6 (14%)
$^1B_1$		9.211920756	0.230293	H-6->L+1 (23%), H-4->L+4 (31%), H-2->L+5 (14%), HOMO->L+7 (14%)
$^1B_2$		9.211952755	0.230312	H-6->L+2 (23%), H-3->L+4 (31%), H-2->L+6 (14%), H-1->L+7 (14%)
$^1A_1$	$^1T_2$	9.400303904	0.029252	H-5->L+4 (56%), H-1->L+5 (21%), HOMO->L+6 (21%)
$^1B_2$		9.400340903	0.029205	H-3->L+4 (56%), H-2->L+6 (21%), H-1->L+7 (21%)
$^1B_1$		9.400350903	0.029189	H-4->L+4 (56%), H-2->L+5 (21%), HOMO->L+7 (21%)

$^1B_2$		10.07177861	0.011647	H-5->L+5 (31%), H-3->L+7 (31%), H-1->L+8 (25%)
$^1B_1$	$^1T_2$	10.07177961	0.011650	H-5->L+6 (31%), H-4->L+7 (31%), HOMO->L+8 (25%)
$^1A_1$		10.07187461	0.011657	H-3->L+5 (31%), H-4->L+6 (31%), H-2->L+9 (34%)
$^1A_1$		10.28358016	0.000636	H-3->L+5 (16%), H-4->L+6 (16%), H-2->L+9 (62%)
$^1B_1$	$^1T_2$	10.28366815	0.000627	H-5->L+6 (16%), H-4->L+7 (16%), HOMO->L+8 (47%), H-1->L+9 (16%)
$^1B_2$		10.28367115	0.000627	H-5->L+5 (16%), H-3->L+7 (16%), H-1->L+8 (47%), HOMO->L+9 (16%)
$^1A_1$		10.50738639	0.019921	HOMO->L+11 (42%), H-1->L+12 (42%), H-10->LUMO (4%), H-3->L+5 (2%), H-4->L+6 (2%), H-7->L+7 (3%)
$^1B_1$	$^1T_2$	10.50759539	0.019929	HOMO->L+10 (42%), H-2->L+12 (42%), H-8->LUMO (4%), H-6->L+5 (2%), H-5->L+6 (2%), H-4->L+7 (2%)
$^1B_2$		10.50759839	0.019929	H-1->L+10 (42%), H-2->L+11 (42%), H-9->LUMO (4%), H-5->L+5 (2%), H-6->L+6 (2%), H-3->L+7 (2%)
$^1B_1$		10.91257996	0.041395	H-6->L+5 (51%), H-7->L+6 (17%), H-3->L+9 (14%)
$^1B_2$	$^1T_2$	10.91258196	0.041396	H-7->L+5 (17%), H-6->L+6 (51%), H-4->L+9 (14%)
$^1A_1$		10.91264096	0.041440	H-5->L+8 (19%),H-4->L+11 (2%), HOMO->L+11 (2%), H-3->L+12 (2%), H-1->L+12 (2%)

Electronic configuration of $\tilde{X}$ $^1A_1$ state in C_{2v} (DFT/PBE0/aug-cc-pVTZ):

Core orbitals: $(1b_1)^2$ $(1a_1)^2$ $(1b_2)^2$ $(2a_1)^2$ $(3a_1)^2$ $(4a_1)^2$ $(2b_1)^2$ $(2b_2)^2$ $(5a_1)^2$ $(6a_1)^2$ $(3b_1)^2$ $(3b_2)^2$ $(7a_1)^2$ $(8a_1)^2$ $(1a_2)^2$ $(4b_2)^2$ $(9a_1)^2$ $(4b_1)^2$ $(5b_1)^2$ $(2a_2)^2$ $(5b_2)^2$

Valence orbitals: $(10a_1)^2$ $(6b_2)^2$ $(11a_1)^2$ $(6b_1)^2$ $(12a_1)^2$ $(13a_1)^2$ $(7b_2)^2$ $(7b_1)^2$ $(14a_1)^2$ $(3a_2)^2$ $(15a_1)^2$ $(8b_1)^2$ $(8b_2)^2$ $(4a_2)^2$ $(9b_2)^2$ $(9b_1)^2$

Unoccupied orbitals: $(16a_1)$ $(10b_2)$ $(10b_1)$ $(17a_1)$ $(18a_1)$ $(11b_2)$ $(11b_1)$ $(19a_1)$ $(20a_1)$ $(5a_2)$

Electronic configuration of $\tilde{X}$ $^1A_1$ state in T_d (DFT/PBE0/aug-cc-pVTZ):

Core orbitals: $(1t_2)^6$ $(1a_1)^2$ $(2a_1)^2$ $(2t_2)^6$ $(3a_1)^2$ $(3t_2)^6$ $(4a_1)^2$ $(1e)^4$ $(4t_2)^6$ $(1t_1)^6$

Valence orbitals: $(5a_1)^2$ $(5t_2)^6$ $(6a_1)^2$ $(6t_2)^6$ $(2e)^4$ $(7t_2)^6$ $(2t_1)^6$

Unoccupied orbitals: $(7a_1)$ $(8t_2)$ $(8a_1)$ $(9t_2)$ $(3e)$

Table S3. Harmonic frequencies from the DFT/PBE0/aug-cc-pVDZ level for carbon tetrachloride neutral electronic ground-state, in the C_{2v} (and T_d) point group, compared with experimental data.

$\tilde{X}\ ^1A_1$								
this work		mode	Exp. [1]		Exp. [2]	Assignment (C_{2v})	mode	Assignment (T_d)
cm^{-1}	eV	(C_{2v})	cm^{-1}	eV	cm^{-1}		(T_d)	
476.4	0.059	$\nu_1(a_1)$	459	0.057	453.7/456.6/459.5/462.7	symmetric C–Cl stretching	$\nu_1(a_1)$	symmetric C–Cl stretching
220.8	0.027	$\nu_2(a_1)$	217	0.027	218 [a]	symmetric C–Cl stretching/deformation	$\nu_2(e)$	degenerate deformation
220.8	0.027	$\nu_3(a_2)$				symmetric C–Cl stretching/deformation		
791.8	0.098	$\nu_4(a_1)$	776	0.096	793.1 / 795.2 797.4 / 790.6	asymmetric C–Cl stretching	$\nu_3(t_2)$	degenerate stretching
791.8	0.098	$\nu_5(b_1)$				asymmetric C–Cl stretching		
791.8	0.098	$\nu_6(b_2)$				asymmetric C–Cl stretching		
322.3	0.040	$\nu_7(a_1)$	314	0.039	313.6 / 309.7	asymmetric C–Cl stretching/ deformation	$\nu_4(t_2)$	degenerate deformation
322.3	0.040	$\nu_8(b_1)$				asymmetric C–Cl stretching/ deformation		
322.3	0.040	$\nu_9(b_2)$				asymmetric C–Cl stretching/ deformation		

[a] from ref. [3]

Table S4. Harmonic frequencies from the DFT/PBE0/aug-cc-pVDZ level for carbon tetrachloride cationic electronic ground-state in the C_1 point group.

$\tilde{X}\ ^2A$			
this work		mode	Assignment
cm^{-1}	eV		
198.1	0.025	ν_1(a)	asymmetric C–Cl stretching
219.2	0.027	ν_2(a)	asymmetric C–Cl stretching
285.1	0.035	ν_3(a)	asymmetric C–Cl stretching
301.8	0.037	ν_4(a)	symmetric C–Cl stretching
315.9	0.039	ν_5(a)	symmetric C–Cl stretching
476.2	0.059	ν_6(a)	C–Cl stretching / deformation
524.2	0.065	ν_7(a)	asymmetric C–Cl stretching
775.0	0.096	ν_8(a)	symmetric C–Cl stretching
929.6	0.115	ν_9(a)	asymmetric C–Cl stretching

References

[1] T.J. Wallington, B.P. Pivesso, A.M. Lira, J.E. Anderson, C.J. Nielsen, N.H. Andersen, Ø. Hodnebrog, CH3Cl, CH2Cl2, CHCl3, and CCl4: Infrared spectra, radiative efficiencies, and global warming potentials, J. Quant. Spectrosc. Radiat. Transf. 174 (2016) 56–64. https://doi.org/10.1016/j.jqsrt.2016.01.029.

[2] T. Chakraborty, A.L. Verma, Vibrational spectra of CCl4: Isotopic components and hot bands. Part I, Spectrochim. Acta. 58 (2002) 1013–1023.

[3] Y. Morino, Y. Nakamura, T. Iijima, Mean square amplitudes and force constants of tetrahedral molecules. I. Carbon tetrachloride and germanium tetrachloride, J. Chem. Phys. 32 (1960) 643–652. https://doi.org/10.1063/1.1730774.
